# Active components and molecular mechanism of *Syringa oblata* Lindl. in the treatment of endometritis based on pharmacology network prediction

**DOI:** 10.3389/fvets.2022.885952

**Published:** 2022-07-22

**Authors:** Xiao-Zhen Wang, Xue-Jiao Song, Chang Liu, Chen Xing, Tong Wu, Yue Zhang, Jing Su, Jing-You Hao, Xue-Ying Chen, Zhi-Yun Zhang, Yan-Hua Li, Yan-Yan Liu

**Affiliations:** ^1^Heilongjiang Key Laboratory for Animal Disease Control and Pharmaceutical Development, College of Veterinary Medicine, Northeast Agricultural University, Harbin, China; ^2^Heilongjiang Animal Disease Prevention and Control Center, Harbin, China; ^3^Harbin Lvda Sheng Animal Medicine Manufacture Co., Ltd., Harbin, China

**Keywords:** endometritis, *Syringa oblata* Lindl. extracts, network pharmacology, active components, TLRs/NF-κB signaling pathway

## Abstract

Antibiotic treatment of endometritis was limited by the inevitable antibiotic residues and risk of bacterial resistance. Therefore, the development of safe and effective strategies for endometritis treatment is urgently needed. *Syringa oblata* Lindl. (SOL) showed great pharmacological potential against endometritis. However, the active components and underlying mechanism of SOL for endometritis treatment remain indeterminate. In our study, the active components and possible molecular mechanism of SOL against endometritis were predicted through computer data mining and biological networks construction. It was predicted that the main active components of SOL were luteolin, kaempferol, oleanolic acid, and rutin, and their anti-endometritis effect was mainly attributed to the TLRs/NF-κB signaling pathway. Furthermore, a green and efficient deep eutectic solvent combined with ultrasound-assisted extraction (DES-UAE) was performed and optimized to obtain high contents of total flavonoid, rutin, and luteolin. The four predicted active components in the SOL extracts were qualitatively and quantitatively analyzed by LC/MS and HPLC. Finally, the pharmacological effects of SOL and active components have been verified by *Staphylococcus aureus*-endometritis models in mice. H&E staining and bacterial load in uterus tissues assays initially validated the pharmacodynamic effects of SOL, and quantitative real-time PCR (RT-qPCR) and ELISA results confirmed that SOL and four active components could ameliorate the uterus injury caused by *Staphylococcus aureus*, the mechanism of action is related to the TLRs/NF-κB signaling pathway.

## Introduction

Endometritis is a major inflammatory disease and is often associated with reproductive failure (1). It not only causes infertility in humans and animals but also leads to economic losses due to unfavorable effects on survival, productivity, and animal welfare (2, 3). It is reported that in some large farms of China, 47.6% of sows were culled due to various abnormalities, of which 41.4% were culled due to endometritis, and even severe endometritis in sow can also cause sepsis and death (4, 5). The causes of endometritis are very complex. Microbial infection, especially *Staphylococcus aureus* (*S. aureus*) infection, has been regarded as the most common and serious pathogenic factor of endometritis (1, 6, 7). It was reported that *S. aureus* could trigger inflammatory diseases by promoting the production of proinflammatory cytokines (8). Currently, antibiotics play an important role in the successful management of *S. aureus-*induced endometritis (9). However, antibiotic treatment is usually accompanied with antibiotic residues, which severely threaten the public health. In addition, long-term and inappropriate use of antibiotics could accelerate the development of bacterial resistance (10, 11). Thus, safe and effective alternatives are urgently needed for endometritis treatment.

Traditional Chinese medicine (TCM) has gained increasing interest from researchers and has been involved in the current endometritis treatment for its excellent anti-inflammatory properties (12, 13). *Syringa oblata* Lindl. (SOL), which belongs to the Oleaceae family plant, has been widely cultivated in Northeast and Southwest China (14). Pharmacological studies demonstrated that the leaves, bark, and flowerbud of SOL could be used to treat various infections, including dampness, acute icteric hepatitis, and heal inflammations (15, 16). Meanwhile, the plant resources of SOL leaves are abundant (17), and using SOL as veterinary medicinal resources presents many economic benefits while minimizing problems associated with waste disposal (18). Therefore, SOL is regarded as a safe and effective alternative for the treatment of endometritis in pigs.

To realize the efficient application of SOL, one of the preliminary and critical steps lies in the accurate and high-efficient extraction of active components. However, to the best of our knowledge, the active components of SOL leaves against endometritis are still unclear. It is well known that TCM possesses various components, and the pharmacological activity was attributed to the synergistic effects of multiple targets and components. This holistic nature of multi-target and multi-component makes it difficult to clarify the active components of TCM. Network pharmacology is a bioinformatics strategy to uncover multi-component drug action and molecular mechanism by constructing networks from the biological level (19). It can illustrate the synergistic effects and potential molecular mechanism of various components by dissecting the various networks involved in the multi-level and intricate interactions (20), and help us understand the effective compounds in SOL that exert anti-endometritis effects. Therefore, the objectives of this work were to clarify the active components of SOL against endometritis using network pharmacology and determine the molecular mechanism. Then, a green deep eutectic solvent (DES) combined with ultrasound-assisted extraction (DES-UAE) was first performed and optimized to realize high-efficacy extraction of predicted components from SOL. In our study, the DES-UAE method was first applied to extract the active compounds from SOL.

**Figure 1:**
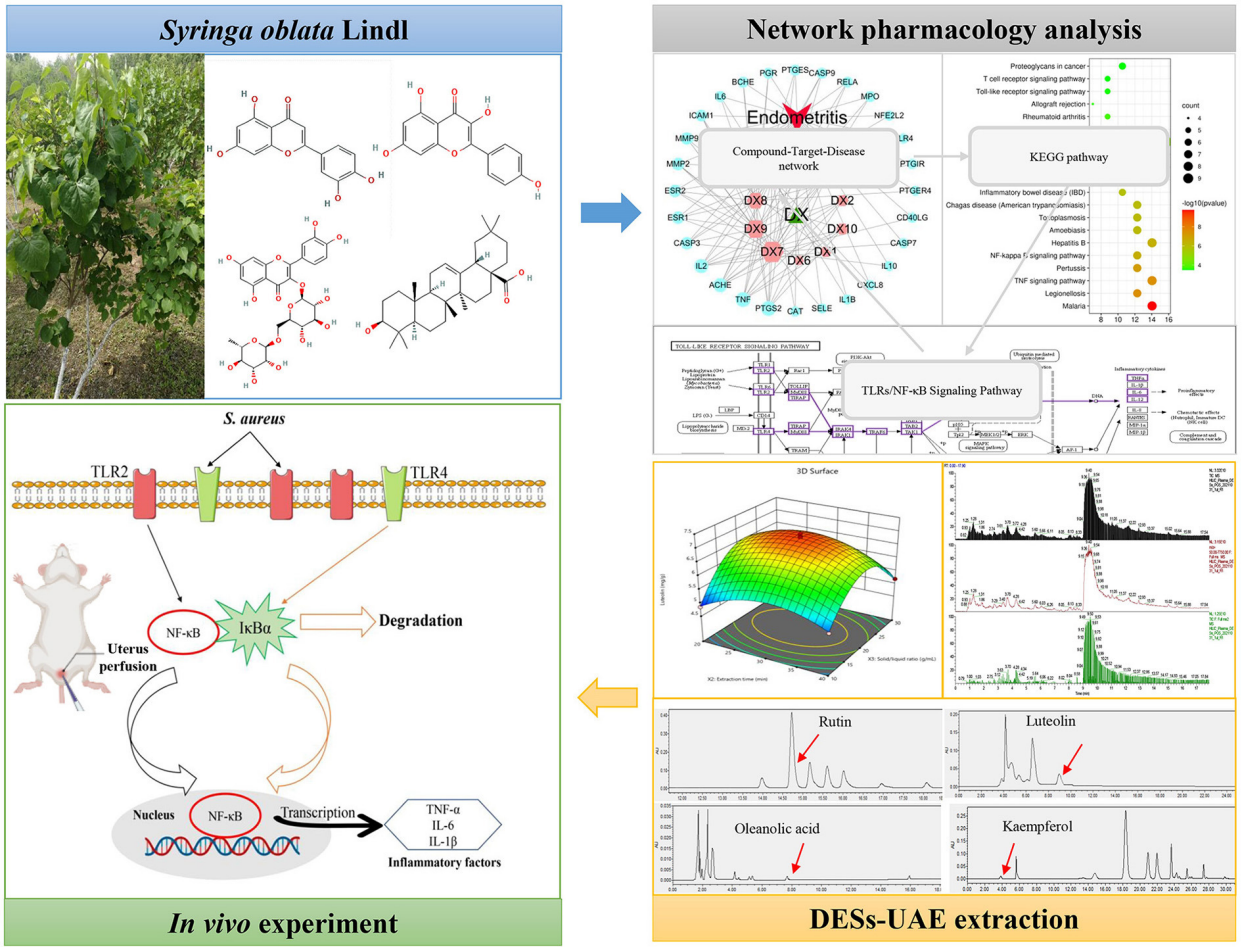
Graphical abstract.

Thus, the purposes of this work were to predict key active components and targets of SOL against endometritis. Then, a green DES-UAE method was first performed and optimized to obtain high contents of underlying anti-endometritis components of SOL. Finally, a mouse model of *S. aureus* induced endometritis was established to elucidate the possible molecular mechanism of SOL against endometritis. Overall, the current study paves the way for the utilization of SOL as a potential source for endometritis treatment and provides a new insight into realizing the efficient application of TCM. The whole workflow is shown in Figure 1.

## Materials and methods

### Network pharmacology prediction

#### Screening of SOL and endometritis targets

Based on previous research (17, 21), 10 components were selected as active compounds of SOL (Supplementary Table S1). The potential targets of SOL were obtained from the TCMSP database (http://tcmspw.com/tcmsp.php) and the Swiss Target Prediction (http://www.swisstargetprediction.ch/). “Endometritis” was used as the keyword, and related disease targets were downloaded from the DisGeNET database (http://www.disgenet.org/), the GeneCard database (https://www.genecards.org/), and the OMIM database (https://omim.org). The gene symbol corresponding to the predicted targets was screened through the Uniprot database (https://www.uniprot.org/), and *sus scrofa* (pig) was selected as the target animal. The Draw Venn Diagram was used to obtain common targets for drug targets and disease targets. Compound–target (C–T) and compound–target–disease (C–T–D) networks were visualized by Cytoscape (version 3.6.1).

#### Protein–protein interaction (PPI) network construction

The common targets associated with SOL and endometritis were imported into the STRING database (http://string-db.org/) to obtain interactions among proteins expressed by target genes; setting the organism as “*sus scrofa*” and the confidence >0.7, the graphical interactions network was constructed and analyzed using Cytoscape (version 3.6.1).

#### Functional enrichment analysis

Gene Ontology (GO) and Kyoto Encyclopedia of Genes and Genomes (KEGG) pathway enrichment analysis were carried out by the DAVID database (https://david.ncifcrf.gov/) (*p* < 0.05), and species were selected as *sus scrofa* (pig). The GO enrichment analysis result was plotted using the GraphPad Prism 8 software, and the KEGG pathway enrichment analysis result was visualized by using bioinformatics (http://www.bioinformatics.com.cn/), an online data visualization software.

### Plant materials and reagents

*Syringa oblata* Lindl. was growing naturally on the campus of Northeast Agricultural University (N 45°44′33.64″, E 126°43′22.07″) in Harbin, Heilongjiang Province of China, and were authenticated by Professor Xiuju Wu from College of Life Sciences, Northeast Agricultural University. The raw materials were dried, pulverized, sifted through an 80-mesh sieve, and stored at 4°C before use. Voucher specimens were deposited at the Department of Animal Pharmacy, Northeast Agricultural University. Rutin, luteolin, oleanolic acid, and kaempferol standards (purity≥98%) were purchased from the Beijing Solarbio Science & Technology Co., Ltd. (Beijing, China). L-proline, L-lysine, 1,2-propanediol, glycerol, glycol, 1,4-butanediol, methanol, and other chemicals were obtained from the Shanghai Aladdin Biochemical Technology Co., Ltd. (Shanghai, China). D101 macroporous resin was procured from the Tianjin BSF Resin Technology Co. Ltd. (Tianjin, China).

### Green extraction of active components

A green and efficient deep eutectic solvent (DES) combined with ultrasound-assisted extraction (DES-UAE) was developed to extract active components of SOL. The detailed extraction method is described in Supplementary Material 1.

### Qualitative analysis of key active compounds of SOL

The main compounds of SOL extracts were analyzed using a Vanquish UPLC system coupled with a Q Exactive^TM^ HF-X Hybrid Quadrupole-Orbitrap^TM^ Mass Spectrometer (UHPLC-HRMS; Thermo Fisher Scientific). The mass spectrometer was operated in negative or positive ion mode. LC separation was done on an ACQUITY UPLC BEH Amide column (2.1 mm × 100 mm, 1.7 μm) using a gradient of solvent A (10 mM ammonium formate, acetonitrile:water = 95:5, and 0.1% formic acid) and solvent B (10 mM ammonium formate, acetonitrile:water = 50:50, and 0.1% formic acid) in positive ion mode, and solvent A (10 mM ammonium acetate, acetonitrile:water = 95:5, and pH = 8) and solvent B (10 mM ammonium acetate, acetonitrile:water = 50:50, and pH = 8) in negative ion mode. The flow rate was 0.3 mL/min, the injection volume was 5 μL, and the column temperature was 25°C. In MS acquisition, the instrument was set to acquire the m/z range of over 70–1,050 with an MS resolution of 60,000. Raw data were collected by mass spectrometry, peak extraction, and retention time using the compound discoverer software (Thermo Fisher Scientific).

### Quantitative analysis of key active compounds of SOL

The contents of rutin, luteolin, oleanolic acid, and kaempferol of SOL extracts were determined using the high-performance liquid chromatography (HPLC) analysis. The samples were filtered through a 0.45 mm membrane filter and loaded for HPLC analysis. The determination was performed on a Thermo C18 column (4.6 mm × 250 mm, 5 μm) at a temperature of 30°C. The information on the mobile phase, UV wavelengths, and injection volumes are shown in Supplementary Table S2.

### Animals

Fifty-five Kunming female mice (6–8 weeks, 32–36 g) were purchased from the Experimental Animal Center of the Second Affiliated Hospital of Harbin Medical University. The research was conducted in accordance with the Institutional Animal Care and Use Committee of Northeast Agricultural University (No. NEAUEC20).

### Establishment of endometritis model in mice and treatment strategy

All animals were first acclimated in temperature (22 ± 3°C) controlled room with 12 h light cycle. All mice were randomly divided into 11 groups (with five mice each): (1) control group; (2) 0.5% CMC-Na group; (3) model group; (4) high-dose SOL-treated (H-SOL) group; (5) low-dose SOL-treated (L-SOL) group; (6) rutin-treated group; (7) luteolin-treated group; (8) oleanolic acid-treated group; (9) kaempferol-treated group; (10) monomer mixture-treated group; (11) DEX-treated group. To induce endometritis, 150 μL *S. aureus* ATCC29213 (1 × 10^6^ CFU/mL) was injected into the uteri of mice *via* the vagina with a blunt needle for 24 h (22). In the SOL-treated group, the mice received 3.13 g/kg and 1.56 g/kg optimized SOL extracts by uterine perfusion. Rutin (24.63 mg/kg), luteolin (21.56 mg/kg), oleanolic acid (1.52 mg/kg), kaempferol (129.91 μg/kg), and monomer mixture (47.84 mg/kg) were given by uterine perfusion and these drugs were dissolved in 0.5% CMC-Na, then the 0.5% CMC-Na was given separately to exclude solvent interference. The dose of monomeric administration was converted according to the content results in SOL extracts. In addition, 5 mg/kg dexamethasone (DEX) was administrated as a positive control (23, 24). Twenty-four hours later, the mice were euthanized and the uterus tissues were collected and stored at −80°C for follow-up experiments.

### Hematoxylin and eosin (H&E) staining

Uterine tissues of each mouse were carefully taken and kept in a 50-mL centrifuge tube which was fixed in 4% paraformaldehyde. After that, uterine tissues were dehydrated and embedded in paraffin wax. Then, uterine sections were cut into 4 μm slices and paraffin-embedded samples were de-waxed with xylene and rehydrated with alcohol. Finally, the sections were stained with hematoxylin and eosin (H&E) and examined under an optical microscope. Histology scores were determined in a blinded fashion. A combined score of tissue damage, hyperemia, edema, and inflammatory cell infiltration was determined. The major histopathological indicators were evaluated by tissue damage, hyperemia, edema, and inflammatory cell infiltration (graded 0–3, from normal to severe, including normal, mild, moderate, and severe) (25, 26).

### Colony-forming units (CFU) of uterus assay

The mice were sacrificed after drug treatment for 24 h. The uterine tissues were harvested and homogenized in sterile saline solution (1:10 *w/v*) on ice. Then, the tissue homogenate was serially diluted in sterile PBS. The homogenates and their serial log dilutions were quickly plated onto TSB agar plates and incubated for 12 h at 37°C. Finally, the *S. aureus* numbers of CFUs remaining in each uterus were evaluated, and bacterial colony counts were presented as mean log10 CFU/ uterus (± SD).

### Quantitative real-time PCR

Total RNA from uterine tissue samples was extracted using an RNAprep Pure Tissue Kit (DP431, Tiangen Biotech, Beijing). The concentration and purity of the total RNA were determined by spectrophotometry (NanoDrop 2000, Thermo, USA) at 260/280 nm. Then, reverse transcription of total RNA into cDNA was performed using a reverse transcription kit (Tiangen Biotech (Beijing) Co., Ltd.). The real-time PCR program was: 1 cycle at 95°C for 10 s, 40 cycles at 95°C for 5 s, followed by 55°C for 15 s and 72°C for 30 s. The primers for qRT-PCR are listed in Table 1. The expression levels of *TLR2, TLR4, NF-*κ*B, I*κ*B*α, *TNF-*α, *IL-6*, and *IL-1*β were quantified relative to the expression of *GAPDH* as the endogenous control by the 2^−Δ*ΔCT*^ method.

**Table 1:** The primers for qRT-PCR.

**Gene**	**Primer (5**′**-3**′**)**	**GeneBank**
		**accession on**.
*TLR2*	Forward: CTCCCAGATGCTTCGTTGTTCCC	NM_011905.3
	Reverse: GTTGTCGCCTGCTTCCAGAGTC	
*TLR4*	Forward: TTGCTGCCAACATCATCCAGGAAG	NM_021297.3
	Reverse: ACCAACGGCTCTGAATAAAGTGTCTAG	
*IκBα*	Forward: CTGAAAGCTGGCTGTGATCCTGAG	NM_010907.2
	Reverse: CTGCGTCAAGACTGCTACACTGG	
*NF-κB*	Forward: AGACCCAGGAGTGTTCACAGACC	NM_001365067.1
	Reverse: GTCACCAGGCGAGTTATAGCTTCAG	
*TNF-α*	Forward: CGCTCTTCTGTCTACTGAACTTCGG	NM_001278601.1
	Reverse: GTGGTTTGTGAGTGTGAGGGTCTG	
*IL-6*	Forward: TTCTTGGGACTGATGCTGGTGAC	NM_001314054.1
	Reverse: AGTGGTATCCTCTGTGAAGTCTCCTC	
*IL-1β*	Forward: CACTACAGGCTCCGAGATGAACAAC	NM_008361.4
	Reverse: TGTCGTTGCTTGGTTCTCCTTGTAC	
*GAPDH*	Forward: CAATGTGTCCGTCGTGGATCT	NM_001289726.1
	Reverse: GTCCTCAGTGTAGCCCAAGATG	

### ELIAS analysis

The uterine tissues homogenate was centrifuged at 12,000 rpm for 15 min at 4°C, and then the supernatant was collected. Biochemical estimations of TLR2, TLR4, NF-κB, IκBα, TNF-α, IL-6, and IL-1β were performed using ELISA kits (Shanghai Enzyme-linked Biotechnology Co., Ltd.), according to the manufacturer's instructions. Briefly, a solid-phase antibody is made by coating the microtiter plate with purified mouse target antibody, adding samples to the coated microtiter wells in sequence, and then combining it with HRP-labeled detection antibody to form an antibody–antigen enzymatic complex, which is thoroughly washed and then colorized with the substrate TMB, which is converted to blue by HRP enzyme and transformed by the action of an acid to the yellow color. Absorbance was observed using an automatic enzyme standard instrument at a test sample of 450 nm. All absorbance results were normalized *via* standard curves.

### Statistical analysis

All data were shown as mean ± standard deviation (SD). Data analyses were carried out using a student's *t-*test, and a value of *p* < 0.05 was considered statistically significant. The statistical analyses were performed using the SPSS 11.0 software (IBM, USA), and pictures were done using the GraphPad Prism 8 software.

## Results

### Potential active compounds and targets of SOL against endometritis

**Figure 2:**
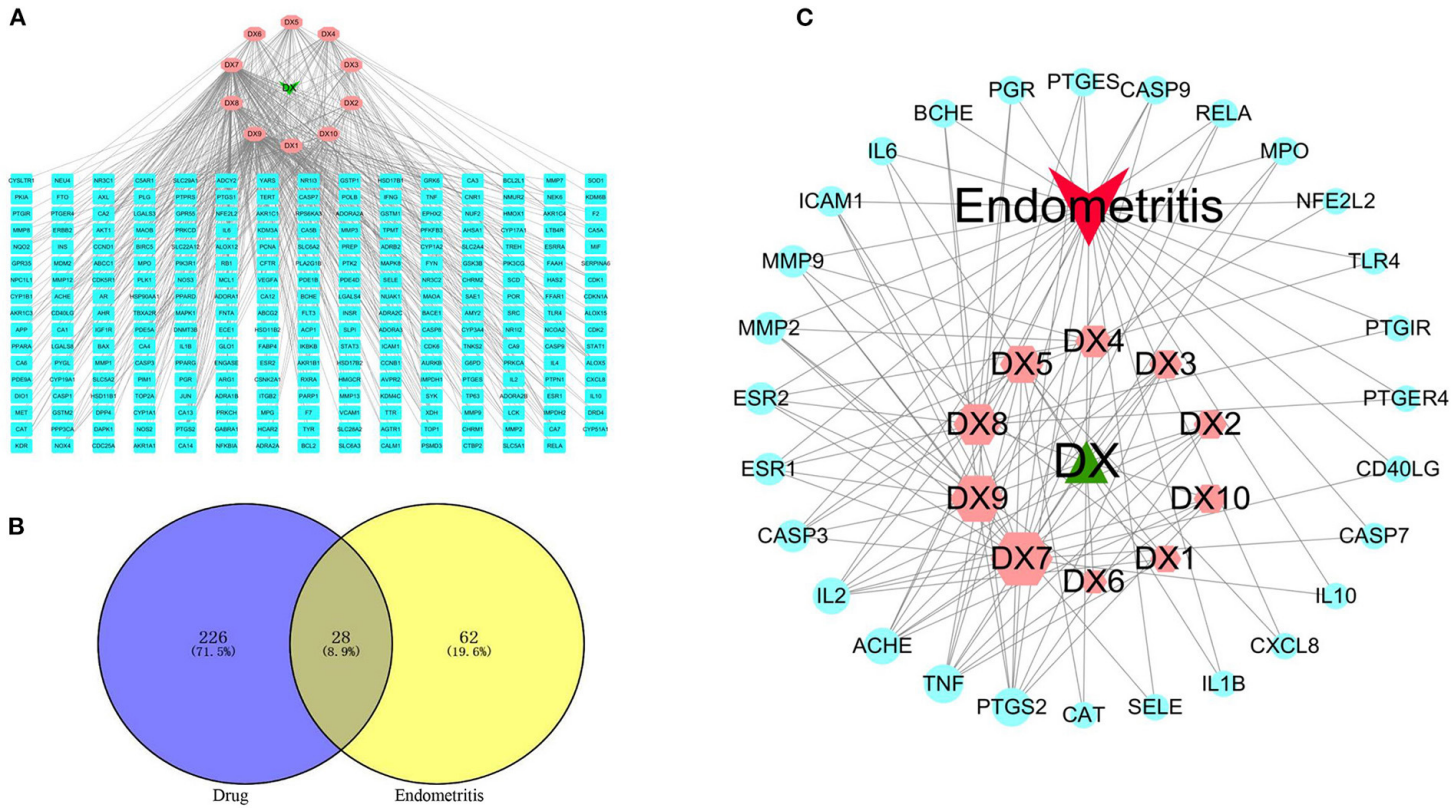
Key active components prediction for SOL treatment of endometritis. **(A)** Compound–target network. The green node represents SOL, the pink nodes represent candidate active components, and the blue nodes represent the targets of SOL components. The lines represent the interactions between them. **(B)** The common targets of SOL and endometritis were identified. **(C)** Compound–target–disease network. The green node represents SOL, the red node represents endometritis, the pink nodes represent candidate active components, and the blue nodes represent common targets. The lines represent the interactions between them. A large size represents a higher degree.

A total of 254 SOL targets were screened, and then the C–T network was constructed. As shown in Figure 2A, kaempferol (degree = 140), luteolin (degree = 135), and oleanolic acid (degree = 65) were connected to the most targets, indicating that these three compounds are important active components in SOL. In addition, 90 endometritis-related targets were obtained, and 28 common targets of SOL and endometritis were screened (Figure 2B). The C–T–D network results showed that the active components in SOL with more targets were luteolin (degree = 18), kaempferol (degree = 15), oleanolic acid (degree = 11), and rutin (degree = 10), indicating that these four active components are the main components that exert an anti-endometritis effect (Figure 2C). Therefore, in the next *in vivo* experiments, we will further evaluate the pharmacological effects of these four active components on endometritis. The detailed target information is shown in Supplementary Table S3.

### Protein–protein interaction (PPI) network construction

**Figure 3:**
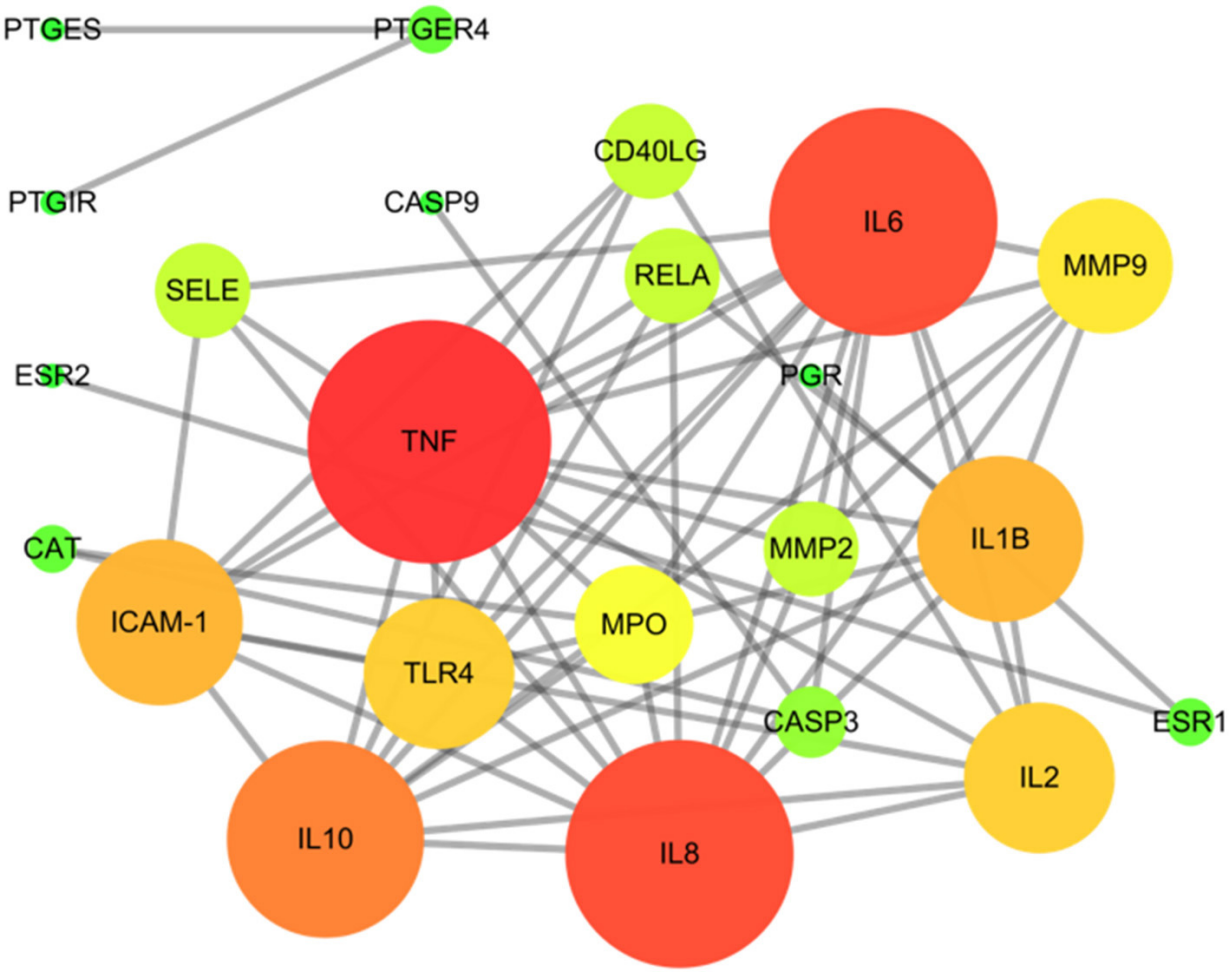
Protein–protein interaction (PPI) network of SOL in treating endometritis. Different colors (from red to green) and the size (from large to small) indicate the degree.

This study is based on the STRING database to analyze interactions between targets of SOL for the treatment of endometritis. The 28 interaction targets of SOL against endometritis were imported into the Cytoscape software for analysis. As shown in Figure 3, the size and color of the node were positively correlated with the Degree value; the larger the node, the larger the Degree value corresponding to the color change from green to red, indicating that this target was more important in this network. In the interaction network, tumor necrosis factor (TNF, Degree = 13), Interleukin-6 (IL-6, Degree = 12), Interleukin-8 (IL-8, Degree = 12), and Interleukin-10 (IL-10, Degree = 10) were at the heart of the direct target of action in SOL treatment of endometritis and may play an important role in SOL against endometritis.

### GO enrichment and KEGG pathway analysis of SOL for endometritis

**Figure 4:**
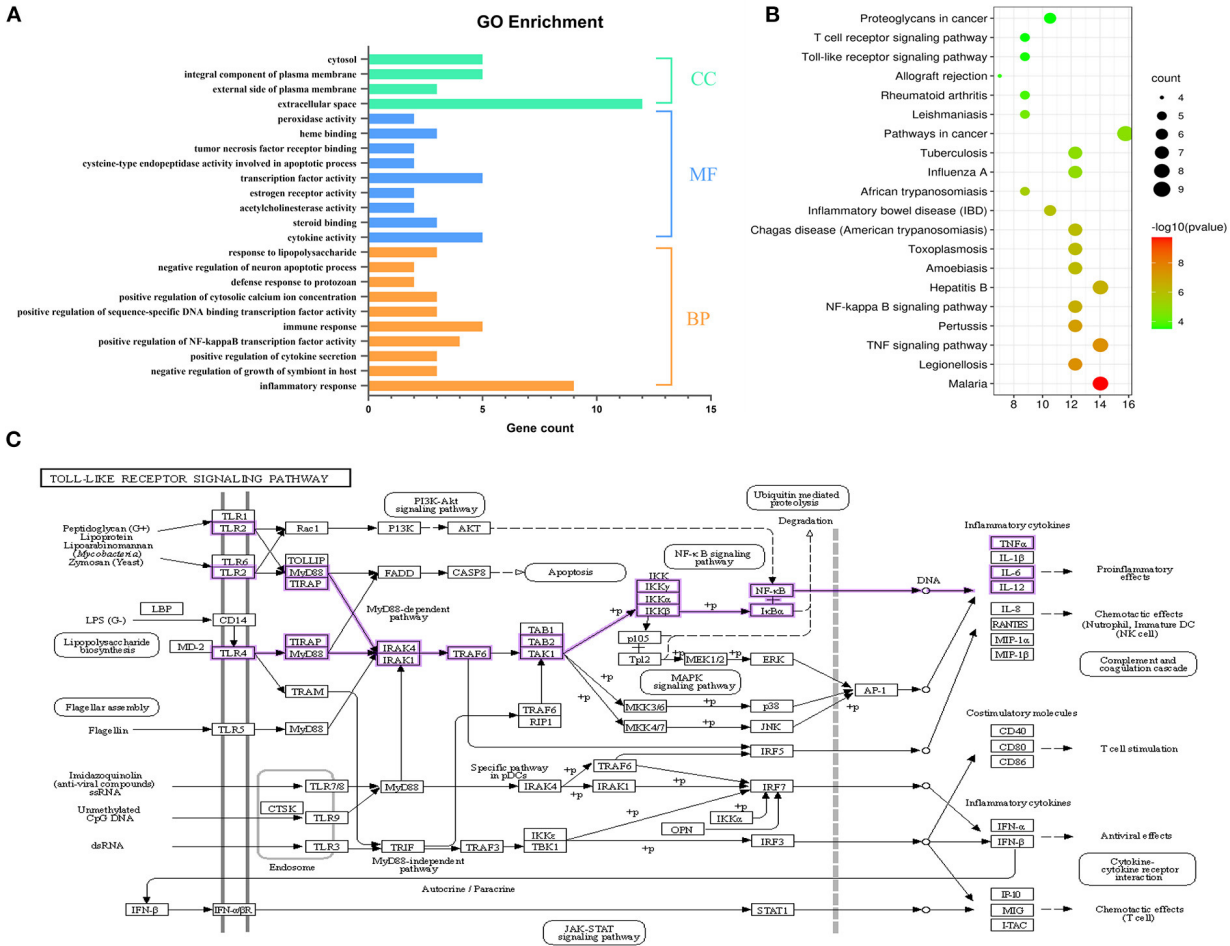
Functional enrichment analysis. **(A)** GO annotation of SOL components acting on the target of endometritis. **(B)** The top 20 KEGG pathway enrichment analysis of key targets. The color scale indicates the *p*-value, and the dot size represents the gene count in each term. **(C)** Pathway diagram of Toll-like receptor signaling pathway.

Using the DAVID database for the core targets related to active components and disease, the threshold value of *p* < 0.05 was set to filter the top biological progress, and then GraphPad Prism 8.0 was used for plotting. Gene Ontology (GO) is the *de facto* standard for gene function description and was widely used in functional annotation and enrichment analysis (27). It includes three branches: biological process (BP), molecular function (MF), and cellular component (CC). As shown in Figure 4A, the items of BP annotations included inflammatory response, immune response, positive regulation of NF-kappa B transcription factor activity, and so on. For MF, the targets were enriched in cytokine activity, transcription factor activity, steroid binding, etc. CC analysis showed more targets were enriched in extracellular space, an integral component of plasma membrane and cytosol. It is reported that the innate immune system was activated when bacterial infestation and endometrial cells secreted large amounts of cytokines and chemokines in response to the inflammatory response (28, 29). The above BP or MF can infer the pathogenic endometritis.

We further conducted a KEGG pathway analysis on the 28 common targets shared by SOL active components targets and endometritis-associated targets. The KEGG functional enrichment totally identified 43 signaling pathways. The top 20 KEGG pathways are shown in Figure 4B; they mainly consisted of four signaling-related pathways associated with inflammation, including TNF signaling pathway, NF-κB signaling pathway, Toll-like receptor signaling pathway, and T cell receptor signaling pathway. Toll-like receptor (TLR) signaling is a central component of the primary innate immune response to pathogenic challenges (30). TLR2 and TLR4, members of the TLR family, are highly expressed in the endometrial cells of the uterus (31, 32). In this study, *S. aureus* was used to induce endometritis in mice, and it has been shown that *S. aureus* could be recognized by TLRs; following TLRs recognition, the NF-κB signaling pathway was activated (33, 34). In view of this, TLRs/NF-κB signaling pathway was selected as the validation pathway (Figure 4C).

### Optimization of SOL extracts

**Figure 5:**
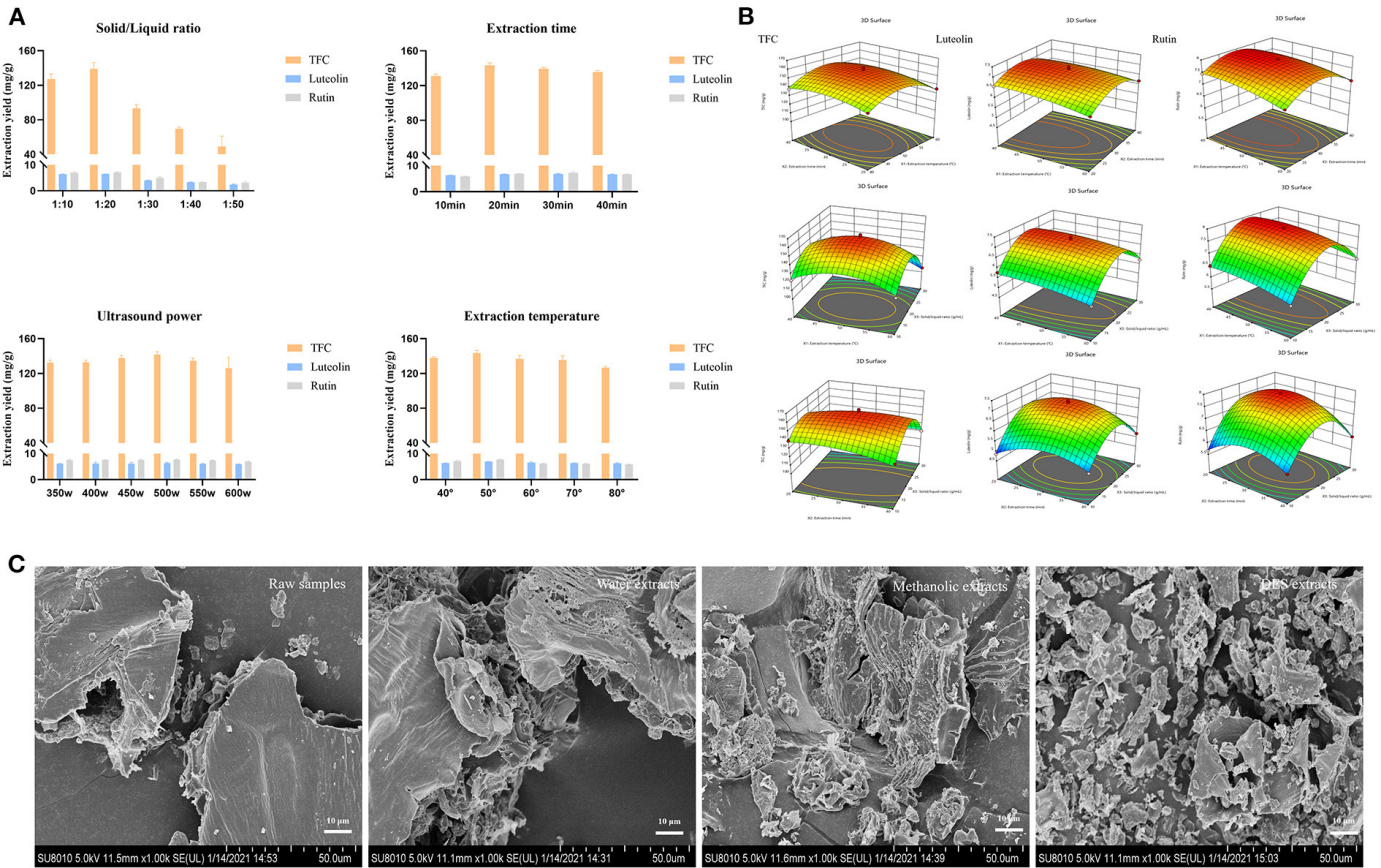
Optimization of SOL extracts. **(A)** Single-factor experiments of solid/liquid ratio, extraction time, ultrasound power, and extraction temperature for TFC, luteolin, and rutin. **(B)** Response surface plots (three-dimensional) showing the effect of the extraction temperature (X1, °C), extraction time (X2, min), and solid/liquid ratio (X3, g/mL) on the extraction yield of total flavonoids (Y1, mg/g), luteolin (Y2, mg/g), and rutin (Y3, mg/g) in SOL. **(C)** Microstructure of SOL samples with different extraction solvents (1000 ×).

Of the four potential active components against endometritis, three of them (luteolin, kaempferol, and rutin) are flavonoids. Considering the effect of the quantitative–effective relationship, the extraction yields of total flavonoid contents (TFC), luteolin, and rutin were further optimized. The optimal solvent was composed of L-Proline/1,4-butanediol with 40% water content (Supplementary Figure S1). The single-factor test results indicated that the extraction yield of TFC, luteolin, and rutin were higher than the results of extraction with other parameters at the solid/liquid ratio of 1:20 g/mL, ultrasound power of 500 W, extraction temperature of 50°C, and extraction time of 30 min (Figure 5A).

To optimize DES-UAE parameters, a 17-run BBD with three conditions (solid/liquid ratio, extraction temperature, and extraction time) and three levels (Supplementary Table S4) were carried out, and the extraction yield of TFC, luteolin, and rutin were taken as the responses (Figure 5B). By solving the regression equation, the optimal value of the chosen variable can be obtained. Design-Expert 12 software calculated that the optimal formulation of TFC, luteolin, and rutin under following condition: solid/liquid ratio was 1:21.50 g/mL, extraction for 33.30 min at 44.40°C, with corresponding Y1 = 151.51 mg/g, Y2 = 6.98 mg/g and Y3 = 7.86 mg/g. To confirm this result, the experiment was conducted under the following conditions three times: solid/liquid ratio was 1:22 g/mL, and extraction for 33 min at 44°C, in which the total flavonoid extraction yield, rutin extraction yield, and luteolin extraction yield were 151.08 ± 1.22 mg/g, 6.97 ± 0.05 mg/g and 7.86 ± 0.08 mg/g, respectively. The predicted values matched the actual results, indicating that the optimization parameters were available. In addition, the surface morphology of SOL with different extraction solvents was observed by SEM (Figure 5C). Different solvents have different degrees of damage to plant cells, and the microstructural damage degree is as follows: DES extracts>methanolic extracts>water extracts>raw samples. These results suggested that plant cells were easily destroyed in DESs conditions, largely due to the fact that DESs could promote the dissolution of plant cell wall fibers (35). Therefore, DES can achieve more efficient extraction of target components than conventional solvents, and the DES-UAE provided a green method for extracting the main components of SOL against endometritis.

### Identification of SOL extracts

To identify the compounds of SOL extracts, LC/MS test was performed. As shown in Table 2, a total of eight compounds, including kaempferol, rutin, luteolin, hyperoside, oleanolic acid, quercetin, isoquercetin, and caffeic acid, were identified. Among them, kaempferol, rutin, luteolin, hyperoside, quercetin, and isoquercitin belong to flavonoids; oleanolic acid is a pentacyclic triterpenoid compound; and caffeic acid is classified as a hydroxycinnamic acid. Kaempferol, rutin, luteolin, and oleanolic acid are the main predicted components of SOL that exert an anti-endometritis effect, and the target components can be extracted with the DES-UAE approach. The total ion current chromatograms (TIC) of SOL extracts are shown in Supplementary Figure S2.

**Table 2:** The active compounds identified from SOL extract.

**Compounds**	**RT**	**Chemical**	**Diff**	**Ion**
	**(min)**	**formula**	**(ppm)**	
Kaempferol	6.172	C_15_ H_10_ O_6_	−4.77	(M+H)+
Rutin	7.88	C_27_ H_30_ O_16_	−4.93	(M+H)+
Luteolin	1.28	C_15_ H_10_ O_6_	−4.77	(M+H)+
Hyperoside	3.56	C_21_ H_20_ O_12_	−4.6	(M+H)+
Oleanolic acid	0.959	C_30_ H_48_ O_3_	3.32	(M–H)–
Quercetin	1.93	C_15_ H_10_ O_7_	3.01	(M–H)–
Isoquercitin	5.291	C_21_ H_20_ O_12_	3.61	(M–H)–
Caffeic acid	6.13	C_9_ H_8_ O_4_	4.61	(M–H)–

### HPLC analysis

The contents of rutin, luteolin, oleanolic acid, and kaempferol of SOL extracts after the treatment with D-101 macroporous were determined by HPLC (Supplementary Figure S3). The content of rutin, luteolin, oleanolic acid, and kaempferol were 10.0 ± 0.16 mg/g, 7.98 ± 0.03 mg/g, 485.70 ± 0.12 μg/g, and 41.57 ± 0.07 μg/g, respectively.

### Improvement of uterus morphology and pathology by SOL

**Figure 6:**
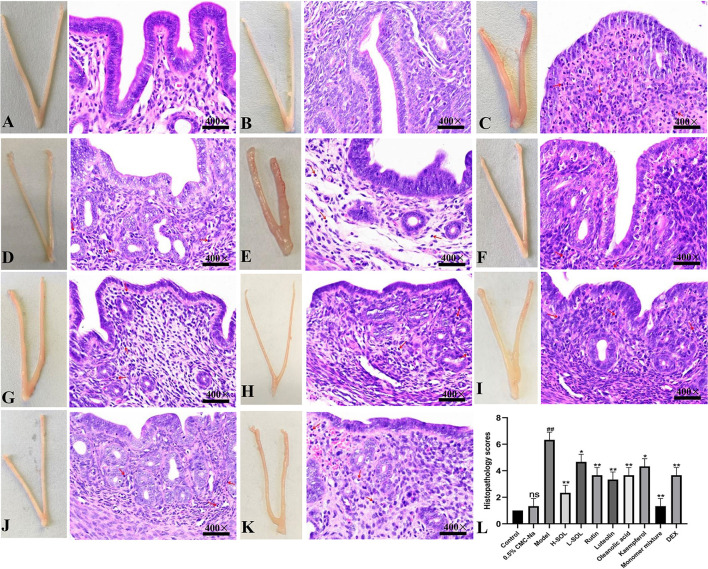
Morphological and histopathological changes. **(A)** Control group. **(B)** 0.5% CMC-Na group. **(C)** *S. aureus* group. **(D)** *S. aureus* + H-SOL group. **(E)** *S. aureus* + L-SOL group. **(F)** *S. aureus* + rutin group. **(G)** *S. aureus* + luteolin group. **(H)** *S. aureus* + oleanolic acid group, **(I)** *S. aureus* + kaempferol group, **(J)** *S. aureus* + monomer mixture group. **(K)** *S. aureus* + DEX group (Hematoxylin and eosin staining, magnification 400 ×, arrows: inflammatory cells). **(L)** Histopathology scores (^*##*^*p* < 0.01, ns *vs*. control group; *p < 0.05, **p < 0.01 *vs*. model group; ns, no significant difference).

In H&E staining, the uterus in 0.5% CMC-Na group had a similar structure to the uterus in the control group (Figures 6A,B). The *S. aureus* group showed severe pathological changes, including endometrial congestion and edema, inflammatory cell infiltration, and decreased number of glands (Figure 6C). The pathological changes caused by *S. aureus* could be attenuated after treatment with SOL extracts, rutin, luteolin, oleanolic acid, kaempferol, and monomer mixture to varying degrees (Figures 6D–J). The degree of inflammatory damage was also slightly reduced in the DEX group (Figure 6K). In addition, tissue in the *S. aureus* group had the highest histological score compared to the control group (*p* < 0.01), and other groups' score was lower than the *S. aureus* group (Figure 6L).

### Bacterial load in uterus tissues

**Figure 7:**
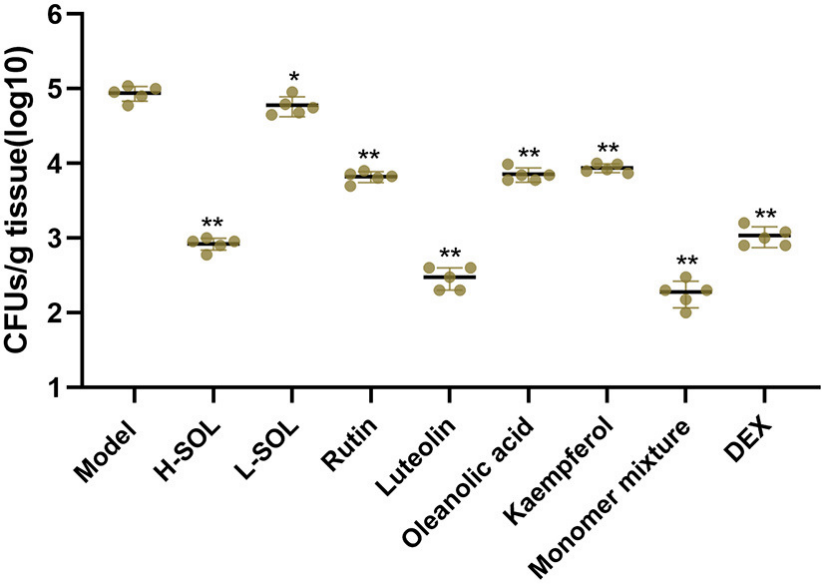
Bacterial load in uterus tissues. The model mice served as control. Results are shown as means ± SD. Significantly different (*p < 0.05 and **p < 0.01) compared to the model group.

In the reproductive tract, the endometrium's initial defense against invasive bacteria depends on the innate immune system (28). Once the innate immune system is activated, endometrial cells secrete large amounts of cytokines and chemokines that can recruit neutrophils and macrophages to clear pathogens (29). As shown in Figure 7, the SOL-treated groups, the monomer groups, the monomer mixture group, and the DEX group could reduce colony counts in uteri *vs*. the model group.

### Molecular expression of the TLRs/NF-κB signaling pathway

**Figure 8:**
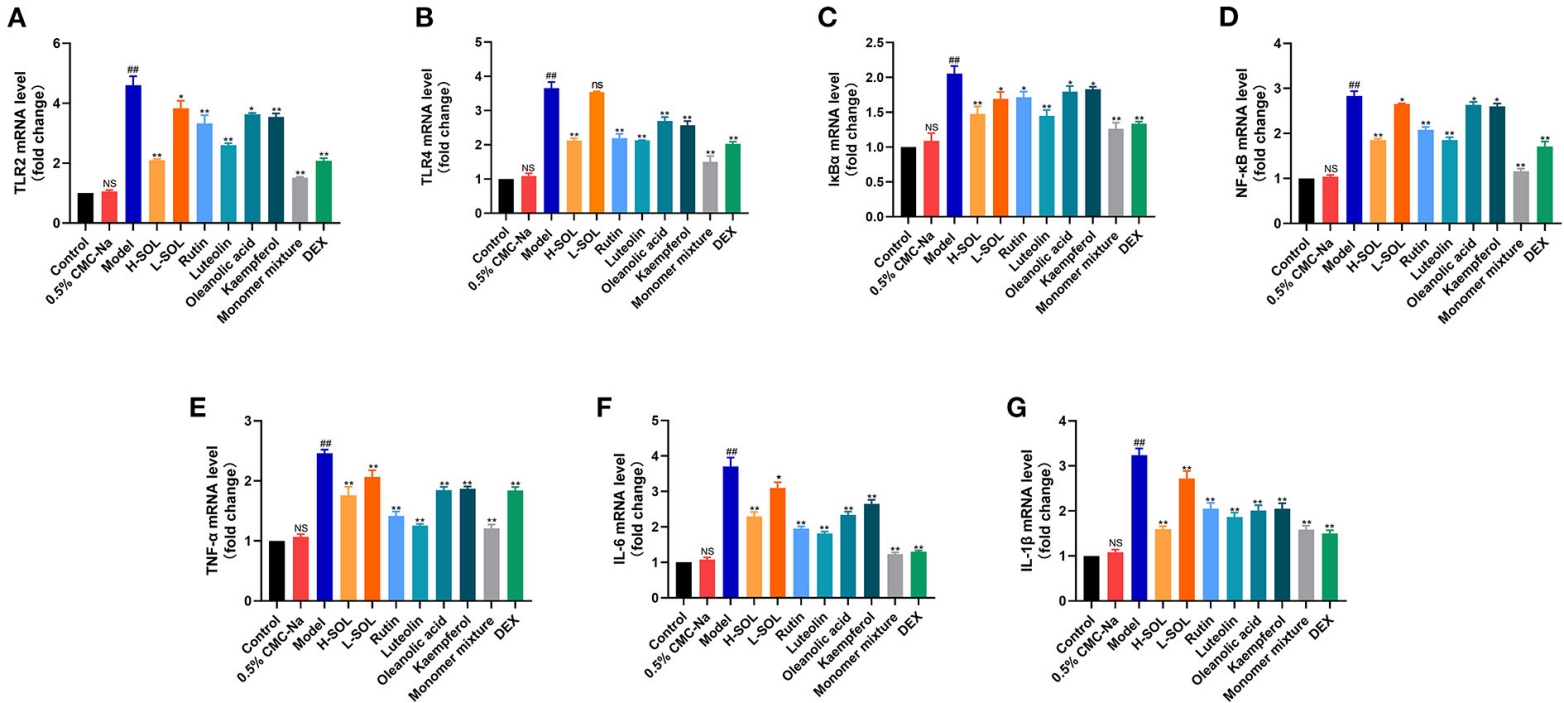
Real-time PCR analysis of *TLR2* **(A)**, *TLR4* **(B)**, *I*κ*B*α **(C)**, *NF-*κ*B* **(D)**, *TNF-*α **(E)**, *IL-6* **(F)**, and *IL-1*β **(G)** transcripts in uterus sample. The relative gene expression level was normalized to the *GAPDH* and reported as relative to the normalized expression in the control group (fold=1). Data are expressed as the mean value ± standard deviation (SD). Statistical analysis is performed with the student's *t*-test. ^##^*p* < 0.01, NS *vs*. control group; *p < 0.05, **p < 0.01 and ns *vs*. model group; NS or ns, no significant difference.

**Figure 9:**
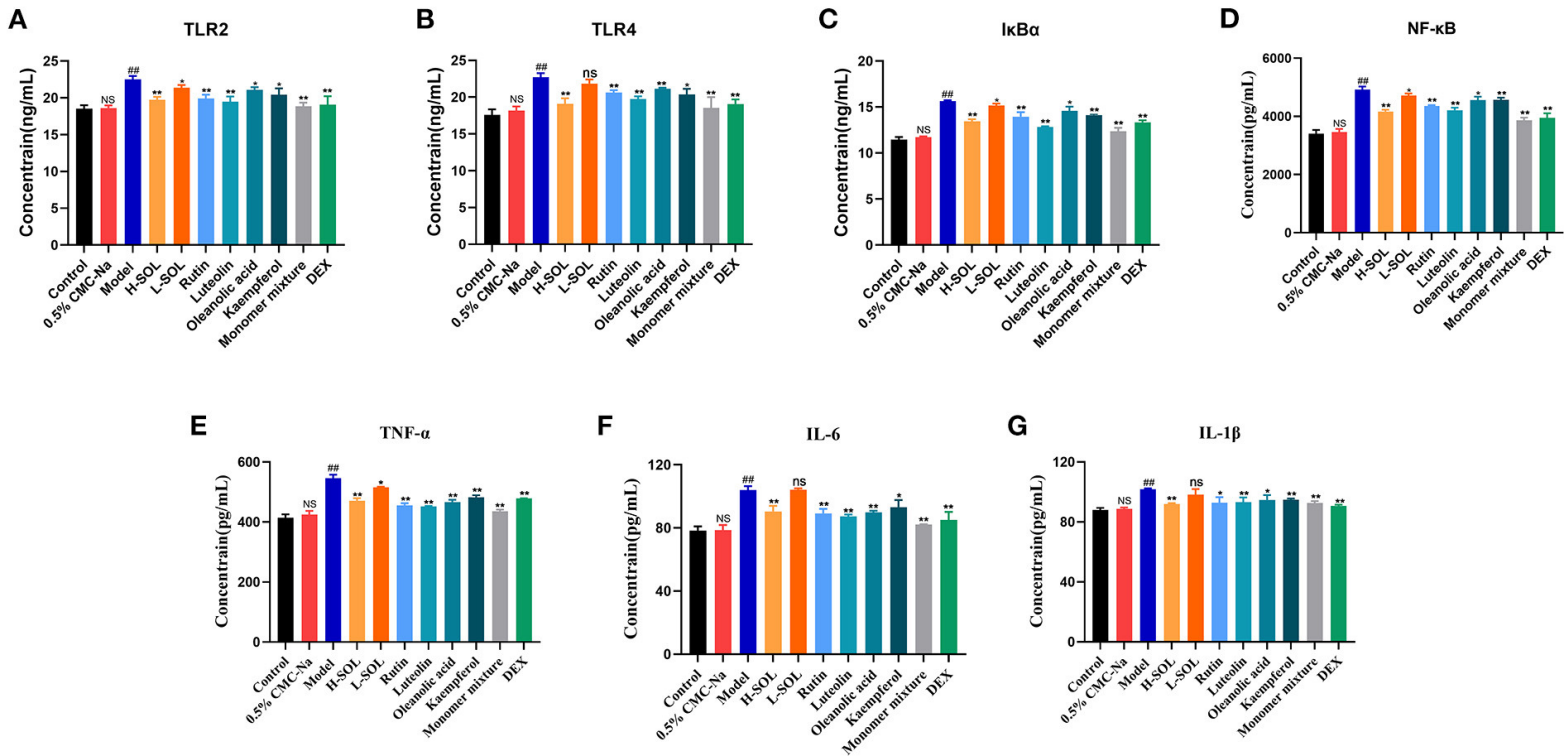
ELISA analysis of TLR2 **(A)**, TLR4 **(B)**, IκBα **(C)**, NF-κB **(D)**, TNF-α **(E)**,IL-6 **(F)**, and IL-1β **(G)** in uterus tissues by activating TLRs/NF-κB pathway. Data are expressed as the mean value ± standard deviation (SD). Statistical analysis is performed with the student's *t-*test. ^*##*^*p* < 0.01, NS *vs*. control group; *p < 0.05, **p < 0.01 and ns *vs*. model group; NS or ns: no significant difference.

To further elucidate the molecular mechanism of SOL against endometritis, we evaluated the regulation of TLRs/NF-κB signaling pathway by SOL. Compared with the control group, the mRNA and protein levels of TLR2, TLR4, IκBα, NF-κB, TNF-α, IL-6, and IL-1β of the model group were significantly upregulated (Figures 8, 9). After drug treatment, *TLR4* mRNA expression, IL-6, and IL-1β protein expression did not show significant differences in the L-SOL group compared with the model group, and other treatment groups downregulated the *TLR4* mRNA expression and IL-6 and IL-1β protein expression compared with the model group. In addition, all drug treatment groups could downregulate the mRNA expression of *TLR2, I*κ*B*α, *NF-*κ*B, TNF-*α, *IL-6, IL-1*β, and protein expression of TLR2, TLR4, IκBα, NF-κB, TNF-α as compared to the model group (Figure 9). These results demonstrated that SOL and predicted active compounds in SOL could alleviate the inflammatory response generated in *S. aureus*-induced endometritis *via* TLRs/ NF-κB signaling pathway.

## Discussion

Endometritis is a common disease in animal production that is considered as the major factor for infertility and economic loss in the sow, equine, and cattle breeding industries (4, 36, 37). So far, multiple targets that accounted for the therapeutic effect of endometritis have been reported. For example, Zhu et al. demonstrated that triggering receptor expressed on myeloid cells-1 deficiency could attenuate the inflammation in mice uteri (38). Zhao et al. showed that the inhibition of NLRP3 inflammasome could inhibit endometritis (26). All these suggest that the treatment of endometritis needs to work through a multi-target, multi-pathway approach. In our previous study, the effect of SOL extracts inhibition of *Streptococcus suis* and *Staphylococcus xylosus* biofilm formation was demonstrated (16, 17). However, the potential application and molecular mechanism of SOL for the treatment of endometritis demand further characterization and identification. Network pharmacology is an emerging field that has been widely used to study the relationship between TCM and disease in a holistic and systematic way (39, 40). Combining multi-target prediction with network analysis, the active components and therapeutic targets of herbal medicines could be clearly understood. Thus, in our study, the network pharmacology combined with *in vivo* validation experiments were used to preliminarily reveal the potential mechanism of SOL against endometritis.

In our study, network pharmacology was performed to decipher the active components and molecular mechanism of SOL against endometritis. The C–T–D network showed that luteolin, kaempferol, oleanolic acid and rutin were ranked in the top four based on network topology parameter (Degree), which play important roles in the treatment of endometritis. Furthermore, 28 potential targets of SOL against endometritis were obtained from 10 compounds of SOL, and most targets were shared by more than two components. These results suggest that most components from SOL affect multiple targets. For example, luteolin, kaempferol, oleanolic acid, and rutin acted on 18, 15, 11, and 10 endometritis-related targets, respectively. Meanwhile, different compounds from SOL may share the same target, demonstrating the synergistic effect between different active ingredients.

PPI networks are viable tools to understand drug design/repositioning, cell functions, and disease machinery (41). Furthermore, the PPI network and GO enrichment analysis get the interaction and biological effect between SOL-endometritis related targets. These targets are closely related to the pathogenesis of endometritis, including inflammatory response, immune response, cytokine activity, etc. It was found that the release of TNF-α, IL-6, and IL-1β plays an important role in the inflammatory response process. Similarly, the effects of TNF-α and IL-1β in the inflammatory response have been reported previously (42). Especially IL-6, which is a pleiotropic cytokine, could regulate multiple biological processes, including immune responses and the development of inflammation. Meanwhile, previous studies have shown that TNF-α, IL-6, and IL-1β were upregulated in *S. aureus*-induced endometritis (43). Thus, the mRNA and protein expression of these three pro-inflammatory cytokines were studied in our study. It was found that the levels of TNF-α, IL-6, and IL-1β were significantly increased upon *S. aureus* infection, which was consistent with previous studies, but after SOL and monomeric components administration, the expression decreased. In addition, the innate immune system of the endometrium was activated to eliminate the invading *S. aureus*. In our study, compared with the model group, all drug administration groups could reduce colony counts in the uteri. The above experimental results are consistent with PPI and GO enrichment predictions. In the KEGG enrichment assay, the importance of the TLRs/NF-κB signaling pathway was identified. Then, the molecular mechanism of SOL against endometritis was further verified based on the *S. aureus-*induced endometritis model in mice. Toll-like receptors (TLRs) are critical in the innate immune response against microbial invasion (44), which could be expressed by the cells of the reproductive system, such as endometrial epithelial cells (45). *S. aureus* stimulation could activate TLR2 and TLR4 (46), and after TLR2 and TLR4 are activated, a downstream signaling NF-κB (p65 and IκBα) is further initiated, including proinflammatory cytokine genes (47, 48). The molecular mechanism diagram of the TLRs/ NF-κB signaling pathway is shown in Figure 4C. The results were also verified *in vivo*. It was found that the mRNA and protein expression of TLR2, TLR4, IκBα, NF-κB, TNF-α, IL-6, and IL-1β were enormously upregulated after *S. aureus* infection. SOL extract and its four components could downregulate the mRNA and protein expression of TLR2, TLR4, IκBα, NF-κB, TNF-α, IL-6, and IL-1β compared to the model group.

Further research is needed for the in-depth development of SOL for endometritis. In our study, a multidisciplinary approach combining bioinformatics (network pharmacology) and *in vivo* pharmacological evaluation was used for the first time to identify active compounds and elucidate the potential molecular mechanism of SOL for the treatment of endometritis. Our preliminary conclusion is that SOL and four main components (luteolin, kaempferol, oleanolic acid, and rutin) could exhibit inflammation inhibition ability and might be involved in the regulation of cytokine activity and immune response to alleviate the pathophysiological process of endometritis from many links. Furthermore, SOL and four main components (luteolin, kaempferol, oleanolic acid, and rutin) could be used for treating endometritis by inhibiting TLRs/ NF-κB signaling pathway. It provides a reference and new platform for scientific research and clinical application of SOL in treating endometritis.

## Data availability statement

The original contributions presented in the study are included in the article/supplementary material, further inquiries can be directed to the corresponding authors.

## Ethics statement

The animal study was reviewed and approved by the Institutional Animal Care and Use Committee of Northeast Agricultural University (No. NEAUEC20).

## Author contributions

Y-HL and Y-YL designed the whole experiment. X-ZW, X-JS, and CL performed the majority of the experiments. X-ZW wrote the manuscript. CX, TW, YZ, JS, J-YH, X-YC, and Z-YZ were supportive during the experiment. All authors have read and agreed to submit this manuscript for publication.

## Funding

This work was supported by the China Agriculture Research System of MOF and MARA and the Key Research and Development Program of Heilongjiang Province (Grant No.GA21B006).

## Conflict of interest

J-YH was employed by Harbin Lvda Sheng Animal Medicine Manufacture Co., Ltd. The remaining authors declare that the research was conducted in the absence of any commercial or financial relationships that could be construed as a potential conflict of interest.

## Publisher's note

All claims expressed in this article are solely those of the authors and do not necessarily represent those of their affiliated organizations, or those of the publisher, the editors and the reviewers. Any product that may be evaluated in this article, or claim that may be made by its manufacturer, is not guaranteed or endorsed by the publisher.
