# Supplementary material for: Active components and molecular mechanism of Syringa oblata Lindl. in the treatment of endometritis based on pharmacology network prediction
Source: Front Vet Sci. 2022 Jul 22;9:885952. doi: 10.3389/fvets.2022.885952 (PMC9355479; doi:10.3389/fvets.2022.885952)

Supplementary Material

**Active Components and Molecular Mechanism of *Syringa oblata* Lindl. in the Treatment of Endometritis Based on Pharmacology Network Prediction**

Xiao-Zhen Wang^1^, Xue-Jiao Song ^1^, Chang Liu^1^, Chen Xing^1^, Tong Wu^1^, Yue Zhang^1^, Jing Su^2^, Jing-You Hao^3^, Xue-Ying Chen^1^, Zhi-Yun Zhang^1^, Yan-Hua Li^1*^ and Yan-Yan Liu^1*^

^1^ Heilongjiang Key Laboratory for Animal Disease Control and Pharmaceutical Development, College of Veterinary Medicine, Northeast Agricultural University, Harbin, China

^2^ Heilongjiang Animal Disease Prevention and Control Center, Harbin, Heilongjiang 150086，P R China

^3^ Harbin Lvda Sheng Animal Medicine Manufacture Co., Ltd., Harbin, China

*** Correspondence:**Corresponding Author: Yanyan Liu and Yanhua Li

E-mail: Liuyanyan@neau.edu.cn and Liyanhua@neau.edu.cn

Supplementary Material 1

**DES-UAE method for SOL extraction**

1. **Preparation of DESs**

A heating and stirring method was used for preparation of DESs. Different HBD and HBA mixtures were heated at 80℃ in round bottom flasks with magnetic stirring for 30-60min until a homogeneous liquid was formed. The water content of samples was firstly determined to reduce the viscosity. Eight different DESs systems were listed in Table 1. The conventional solvents methanol and water were used as controls.

**Table 1:** Different systems of deep eutectic solvents

| Abbreviation | HBA | HBD | Mole ratio |
| --- | --- | --- | --- |
| DES-1 | L-proline | glycol | 1:4 |
| DES-2 | L-proline | 1,2- propanediol | 1:4 |
| DES-3 | L-proline | glycerol | 1:4 |
| DES-4 | L-proline | 1,4- butanediol | 1:4 |
| DES-5 | L-lysine | glycol | 1:4 |
| DES-6 | L-lysine | 1,2- propanediol | 1:4 |
| DES-7 | L-lysine | glycerol | 1:4 |
| DES-8 | L-lysine | 1,4- butanediol | 1:4 |

**2. Experimental design**

To improve active components contents in one of the best *S. oblata* extraction with DES, four parameters such as solid/liquid ratio (1:10 g/mL, 1:20 g/mL, 1:30 g/mL, 1:40 g/mL, 1:50 g/mL), extraction time (10 min, 20 min, 30 min, 40 min), extraction temperature (40 ℃, 50 ℃, 60 ℃, 70 ℃, 80℃) and ultrasound power (350 W, 400 W, 450 W, 500 W, 550 W, 600 W) were performed using single-factor experiments. Based on the results of single-factor experiments, a Box-Behnken design (BBD) was applied to optimize the parameters (solid/liquid ratio, extraction time and extraction temperature) of DES-UAE. The three independent variables including extraction temperature (X1, ℃), extraction time (X2, min) and solid/liquid ratio (X3, g /mL) coupled with three levels were listed in Table 2. The extraction yield of TFC, luteolin and rutin were fitted with the following second-order polynomial model:

$$\text{Y=b}_{0}\text{+}\sum_{\text{i}=1}^{\text{k}} \text{b}_{\text{i}}\text{X}_{\text{i}}\text{+}\sum_{\text{i}=1}^{\text{k}} \text{b}_{\text{ii}}\text{X}_{\text{i}}^{2}\text{+}\sum_{\text{i}<\text{j}} \text{b}_{\text{ij}}\text{X}_{\text{i}}\text{X}_{\text{j}}$$

Where Y is the predicted response function, X_i_ and X_j_ are the independent variables (extraction temperature, extraction time and solid/liquid ratio), and b_0_, b_i_, b_ii_, and b_ij_ are the regression coefficients(constant, linear, quadratic, and interactions terms), as well as k denotes number of variables. A second order polynomial model was obtained using multiple regression of the BBD data using Design-Expert 12. An analysis of variance (ANOVA) was performed to evaluate the model obtained by BBD. In addition, Raw *S. oblata* powders, *S. oblata* extracts with different solvents (DESs, methanol and water), were coated with 10 nm of platinum before the imaging process using SEM (FEI Co. Netherlands) at magnifications of 1000×. The SEM imaging was used to visualize the different extraction solvents on the microstructure of *S. oblata* and then correlate with the extraction efficiency.

**3. Quantification of total flavonoids**

Total flavonoid of *S. oblata* extracts with DESs were determined using aluminum chloride colorimetric method. In short, 50 μL of 5 times dilution of *S. oblata* extract was mixed with 10 μL of 5% *w/v* sodium nitrite solution. After incubation at room temperature for 5 min, 10 μL of 10% *w/v* aluminum chloride was added and incubated for 1 min. Subsequently, 100 μL of 0.5M NaOH solution was added, incubated for 15 min, and finally its absorbance was measured at 510 nm. Total flavonoid content with rutin as standard and all the determinations were carried out in triplicate.

**4. Enrichment of active components from DES extraction solution**

Enrichments of total flavonoid, rutin and luteolin were carried out using D101 macroporous resin. The resins were soaked in anhydrous ethanol for 24 hours and washed thoroughly with pure water, and then dried under vacuum before use. About 10 g of resin and 10 mL of DES extracts obtained under optimised extraction variables were added to the flask, shaken at 120 rpm for 24 h to reach adsorption equilibrium. The DES solvent was removed by washing four times with pure water (50 mL each). Then, 300 mL of 60 % aqueous ethanol (*v/v*) was mixed and shaken continuously at 100 rpm for 24 h to desorb the target compounds. The ethanolic fraction was got and concentrated under vacuum using a rotary evaporator.

Supplementary Material 2

**Supplementary Table S1:** Components in *Syringa oblata* Lindl.

| NO. | Compound | PubChem CID |
| --- | --- | --- |
| DX1 | Syringopicroside | 161619 |
| DX2 | Quercitrin | 5280459 |
| DX3 | Hyperoside | 5281643 |
| DX4 | Caffeic Acid | 689043 |
| DX5 | Rutin | 5280805 |
| DX6 | Chlorogenic acid | 1794427 |
| DX7 | Luteolin | 5280445 |
| DX8 | Oleanolic acid | 10494 |
| DX9 | Kaempferol | 5280863 |
| DX10 | Isoquercitin | 10813969 |

Note: DX, *Syringa oblata* Lindl.; DX1-10, the active components of *Syringa oblata* Lindl.

**Supplementary Table S2:** HPLC analytical conditions.

| Compounds | Mobile phase | Gradient elution | Flow rate | UV wavelength | Injection volume |
| --- | --- | --- | --- | --- | --- |
| Rutin | A: 0.1% formic acid aqueous  B: acetonitrile | 0-30 min: 95%-47% A  30-35 min: 47%-95% A  35-50 min: 95% A | 1.0 ml/min | 355 nm | 20 μL |
| Luteolin | A: 0.2% phosphoric acid water  B: methanol | 0-8 min: 55-62% A | 0.6 ml/min | 350 nm | 10 μL |
|  |  | 8-15 min: 62-70% A |  |  |  |
|  |  | 15-35 min: 70-55% A |  |  |  |
| Oleanolic acid | A: water  B: acetonitrile | A-B (15:85 v/v) | 1.0 ml/min | 210 nm | 10 μL |
| Kaempferol | A: 0.1% phosphoric acid water  B: acetonitrile | 0-15 min: 85-80% A | 0.6 ml/min | 360 nm | 5 μL |
|  |  | 15-25 min: 80-65% A |  |  |  |
|  |  | 25-30 min: 65% A |  |  |  |
|  |  | 30-45 min: 65-50% A |  |  |  |
|  |  | 45-50 min: 50-85% A |  |  |  |

**Supplementary Table S3:** Detailed target information.

|  | Targets |
| --- | --- |
| *Syringa oblata* Lindl-related targets | IL2, HSP90AA1, ADORA1, ADORA2A, ADORA2B, ADORA3, SLC5A2, SLC5A1, TOP1, IMPDH1, IMPDH2, MMP13, MMP1, MMP7, MMP8, LGALS4, SLC29A1, LGALS3, LGALS8, EPHX2, SLC28A2, YARS, PDE5A, MMP9, PARP1, GPR55, AGTR1, CDK2, AKR1B1, CA2, CA7, CA12, CA4, NQO2, ACHE, NOX4, ADRA2C, RPS6KA3, NMUR2, ADRA2A, PTGS2, XDH, TNF, ALOX5, TERT, TOP2A, CYP3A4, HAS2, GSTP1, CA1, CA6, MMP2, CA14, CA9, CA5B, CA5A, CA3, ESR2, HCAR2, MIF, CA13, TLR4, ESR1, SLC6A2, AKR1C4, TPMT, SYK, FYN, LCK, APP, CTBP2, MAOB, PTGS1, KDM3A, KDM6B, FTO, KDM4C, F7, HSD11B1, NFE2L2, STAT3, RELA, IL6, CASP3, POR, SOD1, CAT, IL1B, CXCL8, HMGCR, DIO1, C5AR1, INS, ITGB2, TBXA2R, MMP12, PYGL, PRKCD, PRKCA, NEU4, BACE1, PDE4D, PDE9A, PDE1B, ENGASE, ECE1, KDR, PTGES, CASP7, CASP8, CASP1, DNMT3B, TREH, CDK5R1, MAOA, FLT3, CDK1, GLO1, GSK3B, TTR, CYP1B1, ABCG2, TNKS2, ARG1, PTPRS, ABCC1, CDK6, HSD17B1, HSD17B2, ALOX15, ALOX12, CYP19A1, CSNK2A1, CFTR, GRK6, TYR, AHR, ESRRA, GPR35, DAPK1, AVPR2, IGF1R, F2, PIM1, AURKB, DRD4, MPO, PIK3R1, SRC, PTK2, MMP3, PLK1, MET, NEK6, PLA2G1B, AXL, NUAK1, AKR1C1, AKR1C3, AKR1A1, PFKFB3, PLG, AR, NCOA2, DPP4, PIK3CG, AKT1, VEGFA, CCND1, BCL2L1, CDKN1A, CASP9, MAPK1, IL10, RB1, JUN, TP63, NFKBIA, MDM2, PCNA, ERBB2, PPARG, HMOX1, ICAM1, MCL1, BIRC5, CCNB1, IFNG, IL4, SLC2A4, INSR, CD40LG, NUF2, ADCY2, PTPN1, POLB, ACP1, CDC25A, NOS2, PREP, SCD, FABP4, PPARA, FNTA, FAAH, NR3C1, PPARD, LTB4R, SERPINA6, G6PD, CYP51A1, PGR, HSD11B2, NPC1L1, CYP17A1, PTGER4, SAE1, PRKCH, CNR1, CHRM2, NR3C2, CYSLTR1, PTGIR, FFAR1, NR1I3, SLC6A3, AMY2, RXRA, ADRB2, PKIA, CALM1, MPG, SLC22A12, BCHE, CHRM1, NOS3, ADRA1B, GABRA1, IKBKB, BCL2, BAX, AHSA1, MAPK8, STAT1, CYP1A2, CYP1A1, SELE, VCAM1, NR1I2, PSMD3, PPP3CA, GSTM1, GSTM2, SLPI |
|  |  |
| Endometritis-related targets | PGR, ESR1, OXT, LACTB, SDC1, SELE, CXCL13, TNF, GLUD1, SLC17A5, IL6, IL1B, CXCL8, CD40LG, VDAC1, TLR2, TLR1, TLR6, TLR4, HP, PTGS2, PTGIR, PTGIS, GLS, PLA2G10, GNRH1, PTGES, CXCL6, NLRC5, IFI30, SIGLEC5, GJB2, TIRAP, CNR2, PTGER4, IL13, MMP2, MMP9, NR5A1, ICAM1, ESR2, NR1D1, LEP, IL2, PLK3, IL16, IL17A, CIB1, USF2, TCF21, GPHA2, GGT1, VIM, IL1RN, HIF1A, PRF1, OXTR, IL1A, ABCB7, CCL5, LEFTY2, SST, SELL, IL15, CASP7, CASP6, CASP9, CASP3, MPO, HDAC9, IL10, IFNA1, NLRP3, TLR3, GPAT3, BCHE, RELA, S100A4, SYT1, GORASP1, CAT, PTEN, NUCB2, TXNIP, SLC26A2, FGF12, DDAH2, PRMT1, ACHE, NFE2L2 |
|  |  |
| Common targets | MMP2, TLR4, TNF, CASP9, CAT, MPO, IL10, PTGS2, IL1B, IL2, ESR1, BCHE, CASP7, ICAM1, ESR2, PTGIR, PTGES, ACHE, RELA, CD40LG, IL6, CXCL8, PTGER4, SELE, PGR, CASP3, NFE2L2, MMP9 |

**Supplementary Table S4:** Box-Behnken design matrix with coded and actual variables and results of response

| Run | Factor 1 | Factor 2 | Factor 3 | Response 1 | Response 2 | Response 3 |
| --- | --- | --- | --- | --- | --- | --- |
|  | X1: Temperature | X2: Time | X3: Soild/Liqud ratio | Y1: TFC | Y2: Luteolin | Y3: Rutin |
|  | ℃ | min | g/ml | mg/g | mg/g | mg/g |
| 1 | 50(0) | 40(+1) | 30(+1) | 120.771 | 5.187 | 6.067 |
| 2 | 40(-1) | 40(+1) | 20(0) | 137.861 | 6.298 | 7.147 |
| 3 | 50(0) | 30(0) | 20(0) | 150.351 | 7.012 | 7.895 |
| 4 | 60(+1) | 30(0) | 30(+1) | 105.435 | 5.731 | 6.603 |
| 5 | 50(0) | 40(+1) | 10(-1) | 130.674 | 5.068 | 5.889 |
| 6 | 40(-1) | 20(-1) | 20(0) | 138.415 | 6.569 | 7.447 |
| 7 | 60(+1) | 20(-1) | 20(0) | 135.464 | 6.18 | 7.06 |
| 8 | 40(-1) | 30(0) | 30(+1) | 107.47 | 6.018 | 6.876 |
| 9 | 50(0) | 30(0) | 20(0) | 160.952 | 7.112 | 7.956 |
| 10 | 50(0) | 30(0) | 20(0) | 158.467 | 7.211 | 7.936 |
| 11 | 40(-1) | 30(0) | 10(-1) | 122.333 | 5.776 | 6.478 |
| 12 | 50(0) | 20(-1) | 10(-1) | 139.149 | 4.854 | 5.659 |
| 13 | 50(0) | 20(-1) | 30(+1) | 102.057 | 5.737 | 6.499 |
| 14 | 60(+1) | 40(+1) | 20(0) | 139.438 | 6.189 | 6.998 |
| 15 | 60(+1) | 30(0) | 10(-1) | 120.764 | 5.24 | 6.003 |
| 16 | 50(0) | 30(0) | 20(0) | 158.264 | 7.079 | 7.894 |
| 17 | 50(0) | 30(0) | 20(0) | 160.478 | 6.812 | 7.894 |

Note: the equations for the TFC (Y1), luteolin (Y2) and rutin (Y3) in a second order polynomial was shown as follows:

$$Y_{2}=+7.05-0.1651X_{1}-0.0748X_{2}+0.2169X_{3}+0.07X_{1}X_{2}+0.0623X_{1}X_{3}-0.191X_{2}X_{3}-0.1282X_{1}^{2}-0.6080X_{2}^{2}-1.23X_{3}^{2}$$

$$Y_{1}=+157.70-0.6222X_{1}+1.71X_{2}-9.65X_{3}+1.13X_{1}X_{2}-0.1165X_{1}X_{3}+6.80X_{2}X_{3}-14.54X_{1}^{2}-5.37X_{2}^{2}-29.17X_{3}^{2}$$

$$Y_{3}=+7.92-0.1605X_{1}-0.0705X_{2}+0.2520X_{3}+0.0595X_{1}X_{2}+0.0505X_{1}X_{3}-0.1655X_{2}X_{3}-0.1452X_{1}^{2}-0.6067X_{2}^{2}-1.28X_{3}^{2}$$

**Supplementary Figure S1:** Optimization of DESs systems. The extraction yields of different solvents (A) and water content (B) for TFC, luteolin and rutin.


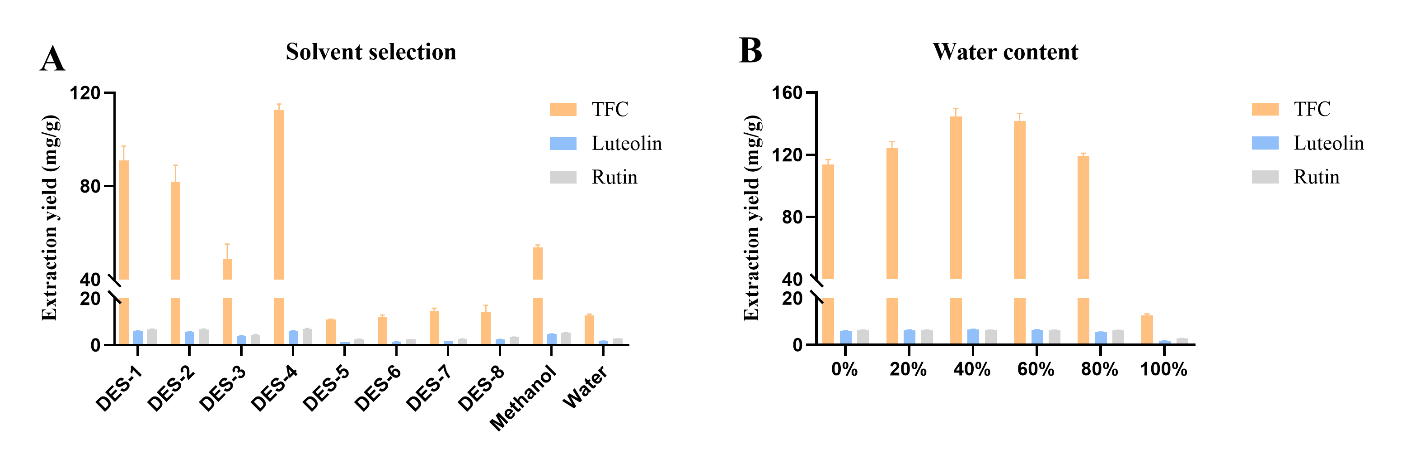


**Supplementary Figure S2:** The total ion current chromatograms (TIC) of SOL extracts.

The chromatograms in positive mode (A) and negative mode (B).


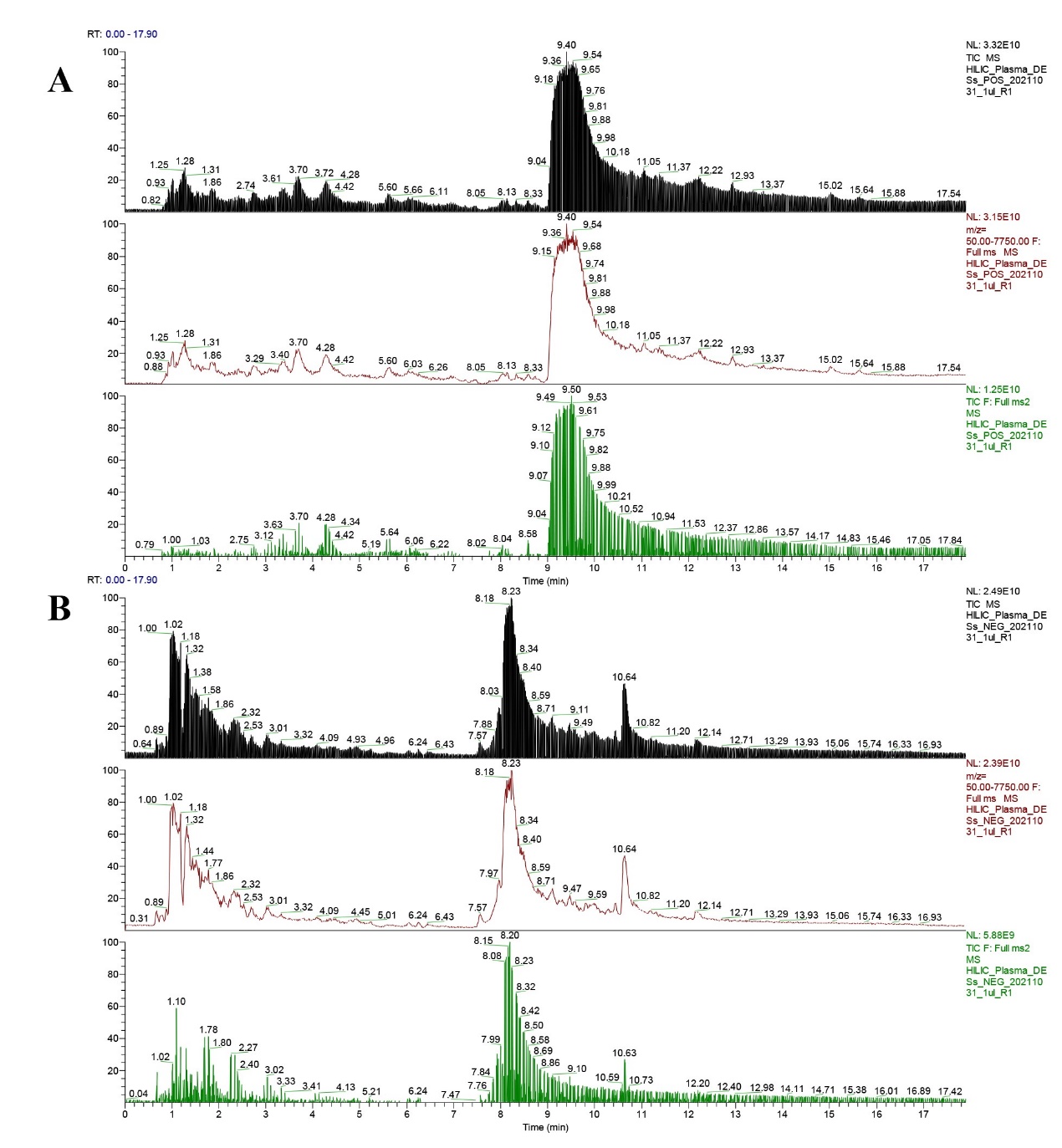


**Supplementary Figure S3:** The content of rutin, luteolin, oleanolic acid and kaempferol in SOL extracts were determined by HPLC. The standard curve of rutin, luteolin, oleanolic acid and kaempferol (A). The liquid chromatogram of rutin, luteolin, oleanolic acid and kaempferol and their corresponding standard (B).


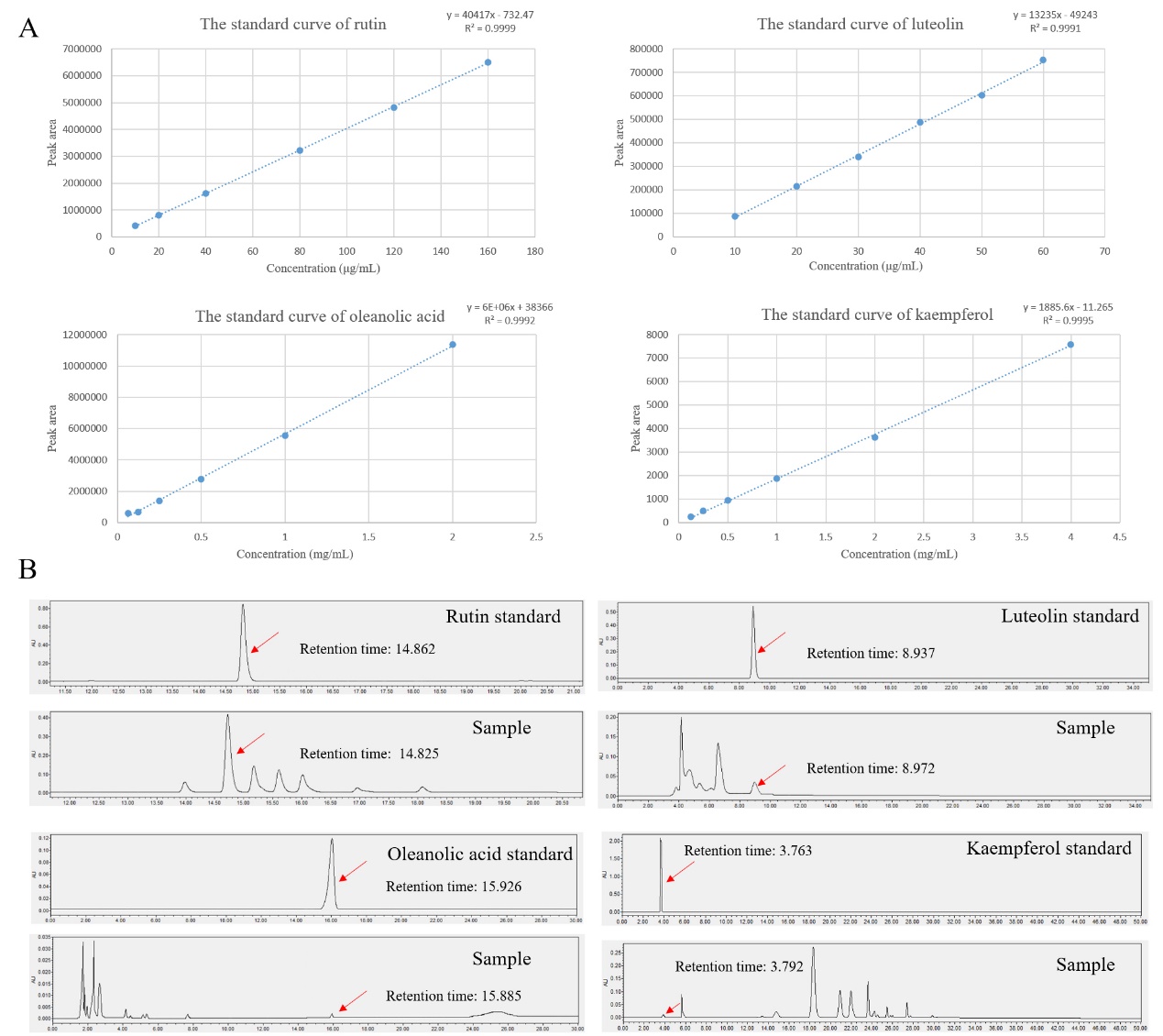

Supplement: Supplementary file 1 [file Data_Sheet_1.docx]
